# The interplay between brain and behavior during development: A multisite effort to generate and share simulated datasets

**DOI:** 10.1038/s41597-025-04740-3

**Published:** 2025-03-21

**Authors:** Neda Sadeghi, Isabelle F. van der Velpen, Bradley T. Baker, Ishaan Batta, Kyle J. Cahill, Sarah Genon, Ethan McCormick, Léa C. Michel, Dustin Moraczewski, Masoud Seraji, Philip Shaw, Rogers F. Silva, Najme Soleimani, Emma Sprooten, Øystein Sørensen, Adam G. Thomas, Audrey Thurm, Zi-Xuan Zhou, Vince D. Calhoun, Rogier Kievit, Anna Plachti, Xi-Nian Zuo, Tonya White

**Affiliations:** 1 Section on Social and Cognitive Developmental Neuroscience, National Institute of Mental Health, National Institutes of Health, Bethesda, Maryland USA; 2 Tri-institutional Center for Translational Research in Neuroimaging and Data Science (TReNDS), Georgia State, Georgia Tech, Emory, Atlanta, Georgia USA; 3 Institute of Neuroscience and Medicine (INM-7), Research Centre Jülich, Jülich, Germany; 4 Institute of Systems Neuroscience, Medical Faculty and University Hospital Düsseldorf, Heinrich-Heine University, Düsseldorf, Germany; 5 Methodology and Statistics Department, Institute of Psychology, Leiden University, Leiden, The Netherlands; 6 Educational Statistics and Research Methods, School of Education, University of Delaware, Newark, USA; 7 Donders Institute for Brain, Cognition and Behavior, Radboud University Medical Center, Nijmegen, The Netherlands; 8 Data Science and Sharing Team, National Institute of Mental Health, National Institutes Health, Bethesda, USA; 9 King’s Maudsley Partnership for Child and Young People, King’s College London, London, UK; 10 Center for Lifespan Changes in Brain and Cognition, Department of Psychology, University of Oslo, Oslo, Norway; 11 Neurodevelopmental and Behavioral Phenotyping Service, National Institute of Mental Health, National Institutes of Health, Bethesda, USA; 12 Developmental Population Neuroscience Research Center, IDG/McGovern Institute for Brain Research, Faculty of Psychology, Beijing Normal University, Beijing, China; 13 Center for the Integrative Developmental Neuroscience, Child Mind Institute, New York, USA

**Keywords:** Computational neuroscience, Statistical methods

## Abstract

One of the challenges in the field of neuroimaging is that we often lack knowledge about the underlying truth and whether our methods can detect developmental changes. To address this gap, five research groups around the globe created simulated datasets embedded with their assumptions of the interplay between brain development, cognition, and behavior. Each group independently created the datasets, unaware of the approaches and assumptions made by the other groups. Each group simulated three datasets with the same variables, each with 10,000 participants over 7 longitudinal waves, ranging from 7 to 20 years-of-age. The independently created datasets include demographic data, brain derived variables along with behavior and cognition variables. These datasets and code that were used to generate the datasets can be downloaded and used by the research community to apply different longitudinal models to determine the underlying patterns and assumptions where the ground truth is known.

## Background & Summary

Neuroimaging has contributed considerably to our understanding of brain development and its relationship to cognition and behavior^1–6^. Magnetic Resonance Imaging (MRI) has enabled us to non-invasively study global and regional brain growth in children and adolescents. With increasing availability of longitudinal neuroimaging studies^7–11^, we can apply statistical models to longitudinal datasets to study the temporal trajectories of development and illness progression^12–15^, as well as the interplay between brain and behavior^16–18^. However, despite advancements in neuroimaging, replicability in research remains a key issue and we lack knowledge of the underlying neurobiology which drives neuroanatomical correlates of cognition and behavior, and their interplay.

Replicability of brain-behavior studies will improve with pre-registration, better study design (e.g. longitudinal studies), better phenotyping, technological advancements that can lead to better data quality and higher signal to noise ratios, increased sample size afforded by large scale studies, data sharing and open science, and cost-effective use of existing research^19,20^. However, one of the major challenges in the field of neuroimaging is that in most cases, we lack knowledge about the underlying truth and whether our methods can detect developmental changes. Simulated datasets represent one way that we can test hypotheses and assess whether our current models can capture complex brain-behavior relationships, and whether our assumptions limit us to certain models.

Here, we focus on the critical period of childhood and adolescence, a time of rapid change in physical, emotional, and intellectual growth. Multiple independent groups (five sites) have created simulated longitudinal datasets in line with how they think brain development takes place, including the interaction between brain, behavior, and cognition. Each group worked independently and blinded to the approaches and assumptions made by the other groups. The generated simulated datasets are now freely available to the research community and can be found at 10.17605/OSF.IO/YJT9P^21^.

In this international collaboration, we invite the research community to explore modeling approaches that capture interrelations between the brain, behavior and cognition using the 15 simulated datasets, totaling 150,000 participants. All five groups submitted simulated datasets that were independently designed to reflect their understanding of typical and atypical neurodevelopment and the interplay between brain and behavior. Researchers across the globe are encouraged to explore different statistical models to determine the underlying assumptions and patterns of neurodevelopment, including the interplay between brain, cognition, and behavior.

There have been studies that take one neuroimaging dataset that is then analyzed by multiple researchers, coined ‘many-analysts’ approaches. Many-analysts papers report that the analytic choices that researchers make can lead to variability in the observed results^22^, even in a single dataset. These analytic choices reflect to a certain extent the assumptions that researchers have about the data and the research question, but may also reflect familiarity with certain analytic methods. As Silberzahn *et al*. stated: “The best defense against subjectivity in science is to expose it. Transparency in data, methods, and process gives the rest of the community opportunity to see the decisions, question them, offer alternatives, and test these alternatives in further research.”^22^ Similarly to many-analysts approach projects^23,24^, we expect to find substantial variability in the reported results from community models, representing the many analysts and their analytic choices. We hope that – similarly to another many-analysts neuroimaging effort – our data and subsequent meta-analytic findings contribute to a better understanding of which factors relate to variability in analyses and assumptions of complex brain and behavioral data^23^.

What is different in the current project, is that the original datasets also reflect many analysts and various assumptions. In addition, unlike data from participants, we will have the ground truth to be able to compare the variability in the reported results, including false-positives and false-negatives. An expectation is that the use of these simulated datasets about neurodevelopment will help inform our research community of our biases and assumptions in the influence of different analytical choices that may be tuned to different assumptions. It is important to recognize some of the limitations of using simulated data, as it may not capture true interindividual variability and the assumptions about the characteristics of noise may not be accurate. Additionally, modeling brain-behavior relationships involves complex interactions between numerous factors, while we are working with a limited set of variables. Ultimately, the quality of simulated data depends on the assumptions we make about reality, which may not always be accurate. However, by involving researchers from diverse backgrounds and geographic locations, we hope to ensure a more comprehensive examination of these assumptions and gain valuable insights into our own biases.

## Methods

Each group with expertise in brain development, neuroscience, and computer science simulated longitudinal data independently, based on their understanding of development and its relationship to brain, behavior, and cognition. We focused on global metrics derived from anatomical MRI, including total gray matter volume and cortical thickness, as these measures have good test-retest reliability^25–27^. Several subcortical regions were also included. Each research group simulated three sets of longitudinal data using the criteria listed below and an extensive data dictionary was provided to each group: 10.17605/OSF.IO/YJT9P^21^.

- **Number of subjects:** 10,000
- **Number of waves/time-points per subject:** 7 (approximately every two years)
- **Age Range:** 7–20 years
  - Wave 1 – 7-8 years-of-age
  - Wave 2 – 9-10 years-of-age
  - Wave 3 – 11-12 years-of-age
  - Wave 4 – 13-14 years-of-age
  - Wave 5 – 15-16 years-of-age
  - Wave 6 – 17-18 years-of-age
  - Wave 7 – 19-20 years-of-age
- **Sex:** 0: male (approximately 50%), 1: female (approximately 50%)
- **Cognitive measures:** IQ (mean = 100, standard deviation = 15)
- **Behavioral measures:** internalizing and externalizing symptoms and attention problems scale based on child behavior checklist (CBCL)
- **Autism diagnosis:** 0: no, 1: yes
- **Brain volume measures:** intracranial volume, total gray matter volume, total white matter volume, hippocampal volume, amygdala volume, and cortical thickness of frontal lobe. The units should be in mm3 for volumetric measures and mm for cortical thickness. Each group determines the starting point and trajectory of each brain measure
- **Parental Education:** 0: less than 12th grade (5.3%), 1: High School or GED (35.4%), 2: Bachelor’s Degree (25.3%), 3: Master’s or higher degree (34%)
- **Attrition/Missing data:** Missing timepoints, no greater than 20%
- **Effect size, noise:** Each group should decide what effect size and amount of noise they would like to include.

Research groups submitted three datasets each to the simulation project GitHub site: (https://socoden.github.io/Simulation/). An automated quality check was performed upon submission to assure that each variable adhered to the guidelines and the data dictionary.

## Description of the Simulated Datasets

Each of the five sites independently generated three datasets. A description of the underlying assumptions, decisions, and approach within each of the groups is provided below. This section can be skipped for those who want to first analyze the data independently, without knowing the developmental assumptions that were used to create the datasets.

## dpn Datasets

The simulation parameters for the “dpn” datasets were derived from multiple sources to ensure the generated data closely reflects real-world developmental patterns. First, in Step A, the correlation matrix of brain phenotypes, along with sex-specific ratios for different phenotypes, was estimated from the preprocessed T1-weighted MRI data of the ABCD Data Release 5.0, specifically from samples that passed rigorous quality control. Second, in Step B, normative growth trajectories for brain phenotypes were obtained from the lifespan brain charts published by Bethlehem *et al*.^28^. For brain phenotypes not explicitly covered in the published charts, such as hippocampal and amygdala volumes, growth trajectories were derived by first integrating features reported in the existing literature and then adjusting the normative model data of subcortical volumes accordingly. These normative growth trajectories were subsequently fitted using 4th-order polynomial functions to estimate the normative model parameters for each phenotype. Third, additional parameters (in Step C), such as measurement noise levels and latent correlations between behavioral measures, were informed by a comprehensive review of current literature.

In Step C, true z-scores for brain phenotypes were simulated using the correlation matrix and Cholesky decomposition, ensuring that the relationships between different phenotypes align with population-level patterns. These z-scores were constrained to fall within a biologically plausible range. Longitudinal trajectories of true z-scores for each individual were determined by sampling quadratic curve parameters from a reasonable range, ensuring that the developmental trends of individual brain phenotypes were realistic. Measurement noise was then added to the true z-scores, and the resulting values were transformed into raw measurements using the normative model parameters. Female brain phenotype values were further adjusted using sex-specific ratios. Additionally, the simulation incorporated correlations between true z-scores of brain phenotypes, demographic variables, true IQ scores (before adding measurement noise), autism diagnosis, and CBCL scores. For example, autism diagnosis scores were modeled based on brain phenotype z-scores, IQ, and random noise, with prevalence rates adjusted to reflect known sex differences. The simulation is repeated three times to generate multiple datasets.

The simulated data underwent rigorous quality control to ensure its validity. Distributions of brain phenotypes, behavioral scores, and demographic variables were validated against expected ranges, and the correlations between variables were carefully examined for biological plausibility. The unique aspects of the simulation framework include, but are not limited to, the integration of normative growth trajectories from the lifespan brain charts, the use of a correlation matrix derived from ABCD data, and the incorporation of sex-specific adjustments for brain phenotypes. These features collectively ensure that the simulated data accurately mirrors real-world developmental patterns and variability, providing a robust foundation for methodological development and validation in brain development research.

## leer Datasets

### Computational framework

The datasets were simulated using R, version 4.1.0 (http://www.r-project.org/) and the lavaan package^29^. The command set.seed() was used to ensure reproducibility.

The data simulation followed a stepwise approach, beginning with the generation of trajectories of the six brain measures, followed by the inclusion of the covariates, and concluding with the addition of the missingness pattern. The dataset was simulated for 30,000 participants across seven waves. Both the raw data and covariance matrix were designed to closely resemble patterns reported in the literature. Specifically, we drew upon findings from the HUBU study (“Hjernens Udvikling hos Børn og Unge”, Brain Maturation in Children and Adolescents) for the brain trajectories^30,31^, and multiple published findings regarding other covariates (see below).

### Brain trajectories

Each brain measure was simulated independently using latent growth curve models with a grouping by sex. The shape of the trajectory was informed by our knowledge of a high temporal density childhood sample (HUBU). Trajectories were either specified as quadratic (with a dominant linear component), or as flexibly specified (using a ‘basis model’ approach^32^) to most closely approximate realistic trajectories. The mean and the variance of both intercepts and slopes, as well as their covariance was also specified according to known brain trajectories. We also fixed the variance at each time point to be constant over time and sex. To evaluate the validity of the simulated trajectories, we visualized the raw data for each brain measure and we fitted latent growth curve models to assess model fit and parameter recovery. The six brain measures were combined into a single model, simulating the intercepts and slopes of all six brain measures simultaneously.

### Covariates

Next, we added the covariates to the simulated dataset based on established relationships in the literature. Correlation patterns between brain metrics and covariates were generated, then reviewed to approximate realistic associations. Each covariate was introduced iteratively, and the covariance matrix was examined at each step to ensure that the relationships between covariates and brain measures aligned with expected patterns. The covariates were computed by weighting brain measures and other covariates by specific factors. Subsequently, variables were transformed to approximate their expected distributions. Sex differences were not explicitly simulated for covariates. Any observed sex differences in covariates emerged indirectly from sex differences in brain measures.

In line with common cohort practices and (relative) stability of the measures, ‘parental education’ and ‘autism diagnosis’ were included only at the first wave. Parental education was simulated to have a small positive correlation with brain measures, whereas autism was designed to exhibit a negative correlation with brain measures^33,34^. IQ and CBCL subscales (externalizing, internalizing, and attention problems) were included across the seven timepoints. We chose to simulate their baseline value through weighted sums of the six brain measures as well as specific covariates and add random values from a normal distribution at each time point. IQ was computed to be weakly, but significantly positively correlated with the six brain measures and moderately negatively correlated with parental education^35,36^. Given the relative stability of IQ over time, the other time points were calculated by adding small variation from a normal distribution^37^.

The CBCL externalizing subscale was negatively correlated with brain measures, parental education, and autism diagnosis^38–40^. As externalizing behaviors typically decline during childhood and adolescence^41^, subsequent time points were generated by subtracting a random value drawn from a normal distribution. The internalizing and attention subscales were modeled the same way, while also accounting for their high positive correlation with the externalizing subscale^42^. The internalizing subscale was simulated to increase over time, while the attention subscale was also set to increase over time, following expected trajectories^41^.

Once the covariance matrix was deemed realistic (i.e., mostly in line with reported associations) and unproblematic (no impossible (co)variances or estimation problems), we added date of birth, brain-behavior measurement dates, and age at each wave. The dataset was cleaned by ensuring correct variable types and factor levels. Data were then converted to long format and split into three separate datasets of 10,000 participants each.

### Missingness

Finally, we created the missingness pattern. Missingness was generated at both the time point (i.e., visit drop out) and individual measure (e.g., non-compliance, interrupted measurement) level. The probability of time point drop out increased across subsequent waves, reflecting common data collection conditions. Background characteristics were modeled as increasing (e.g., male, lower parental education, increased psychopathology symptoms) or decreasing (e.g., female, higher parental education) the log-odds of time point drop-out (binary condition, 1 = drop out). MRI measures were subject to increased measure-specific drop out with a negative exponential function over waves, concentrating MRI measure drop out in younger ages. Increased underlying psychopathology symptoms increased the log-odds of measure-specific drop out in psychopathology measures – creating severe non-ignorability in the measures as the trait being measured determines in-part if the measure is included. All log-odds were converted to probabilities and drop out was applied stochastically using those probabilities for each measure at each time point.

## OSA Datasets

Simulation of datasets was performed with R (v. 4.0.0) and the *simstudy* package (v. 0.4.0). Splines for simulated developmental curves were estimated with *splines* package (v. 4.0.0). For data definition, generation and visualization several other packages were used (*mvnfast, truncnorm, lubridate, sn, faux, ggplot2)* to model distributions and relationships between variables. All random processes were controlled using predefined seed values to ensure reproducibility.

First, we defined a cross-sectional dataset with demographic, cognitive, and behavioral variables. Key variables included sex (approximately 50% male, 50% female), age at different assessment waves (7-20 years), parental education (categorized into four levels), autism diagnosis (binary; prevalence not more than 2%, with higher prevalence in males than females). IQ was modeled as following a truncated normal distribution. Behavioral measures, including externalizing and internalizing symptoms and attention problems, were generated based on the literature and considering their relationship to sex and autism diagnoses. To introduce correlations among behavioral traits, we predefined a correlation structure, approximating real-world associations among CBCL (Child Behavioral Checklist) scores.

The datasets were then transformed into a longitudinal format, generating multiple waves of observations per participant, and age was incremented across waves simulating variability.

Brain measures were simulated using spline functions to model age-dependent growth trajectories, with separate parameters for males and females. We incorporated correlations between ICV, hippocampal volume, parental education, and cognitive ability. Additional age-dependent modifications were applied to hippocampal volume trajectories for individuals with autism.

## paint Datasets

Similar to the “dpn” datasets, the “paint” datasets were also generated using the ABCD data, Lifespan growth charts, and relevant literature to simulate data that closely resembles real-world data. First, cross-sectional data was created by sampling from the ABCD Data Release 5.1 to capture correlations between demographic and brain phenotype information. A binomial distribution was applied to randomly assign 3% of the sample as having autism. Next, second-order polynomials were fitted to the normative growth trajectories for brain phenotypes from the lifespan brain charts^28^ using sex, log(age), log(age)^2 as covariates. For brain phenotypes not directly included in these charts, such as hippocampal and amygdala volumes, growth trajectories were developed by utilizing existing literature and adjusting the normative subcortical volume data accordingly. For generating longitudinal data, linear mixed effects models were then applied, where the coefficients for fixed effect parameters were estimated based on the fit to the growth charts, and random effects for each subject were calculated using the subject’s z-score from the ABCD data. Finally, random noise was added for each subject and data point.

Additionally, specific patterns were incorporated into each dataset. For the paint_data1 dataset, we adjusted the subsequent wave data for white matter brain volume and CBCL attention scores so that changes in brain volume predicted changes in the CBCL attention score. In this dataset, we applied a “missing completely at random” approach, assigning 10% of the data to have missing values for the brain phenotypes.

For the paint_data2 dataset, we embedded a relationship between amygdala volume trajectories between waves 1 and 3, coupled with white matter volume and ICV at these waves such that a 100% prediction rate for ASD could be achieved. While this level of accuracy would be very difficult to identify using standard group level analyses, the question was whether machine learning approaches would be able to extract this relationship.

For the paint_data3 dataset, we aimed to simulate a sensitive period in development by embedding a pattern where changes in behavior predicted changes in frontal lobe gray matter thickness between waves four and six. Additionally, in this dataset, we introduced missingness by associating being male and having lower parental education with a higher likelihood of dropping out of the study.

Finally, we ensured that all variables included in these datasets fall within a reasonable range and align with existing literature.

## site4802 Datasets

The simulated data was generated according to a conditioned Gaussian distribution based on the mean and covariance learned from real training data. The data variables were divided into static variables and dynamic variables based on whether they were expected to change across wave numbers in the dataset. Static variables included sex, autism diagnosis, parental education, and IQ, while the dynamic variables included brain measures (total gray matter volume, white matter volume, hippocampal volume, amygdala volume, frontal lobe thickness, intracranial volume) and behavioral assessments (CBCL internalizing and externalizing scores).

The static variables were simulated using random generation as follows. For each subject in the simulated data, sex and autism diagnosis were assigned randomly with uniform probability. Parental education levels were generated using random sampling with a probability of (0.05, 0.35, 0.25, 0.35) for levels (0,1,2,3). IQ score values were generated randomly from a normal distribution (mean = 100, std = 15). The age values were generated using uniform random sampling from a two-year window for a given wave number. The static data along with age was generated for each of the seven waves as per the requirements.

For a given age and the values of static variables, the dynamic variables were generated by random sampling from a Gaussian distribution conditioned on these values. The mean and covariance matrix for this distribution were inferred from real data. The mean of this distribution was computed using a mixed effects polynomial with respect to age, along with interaction terms for static variables. The coefficients of the polynomial were learned by fitting it on the ABCD dataset with real values for all variables^10^. Once the polynomial is learned from the real data, the mean values for the dynamic variables for the simulated data were generated using the entries from the age and static variables from the simulated data. The covariance matrix for the Gaussian distribution was learned from the sample covariance of dynamic variables from the real data.

After having generated the simulated data in the aforementioned manner, two versions of the data were created in addition to the originally simulated data. The second version involved creating random attrition with up to 10% missing data for the entries as described in the simulation criteria, along with additional perturbation to the covariance matrix by adding Gaussian noise (std = 0.2) for entries with correlation of 0.8 or lower. The third version included the same covariance perturbation, along with an additional polynomial effect based on the mean trajectories based on autism diagnosis. The polynomial effect was added such that the level and steepness of the polynomial curve with age for subjects with autism was higher for behavioral measures, and lower for the brain measures compared to subjects without autism, thus making the polynomial trend more pronounced for autism diagnosis.

## Data Records

The dataset is available at Open Science Framework (OSF), 10.17605/OSF.IO/YJT9P, publicly offered as data.zip under CC-BY 4.0 license^21^. The data.zip file contains 15 csv files, three simulated datasets from each site. Each subfolder under the data folder corresponds to the name of the site that generated the data. The site names are the following: dpn, leer, OSA, paint, and site4802. Table 1 shows the folder structure and size of each file.

**Table 1:** Folder structure and size of each file.

data/site4802/site4802_data1.csv	11.6MB
data/site4802/site4802_data3.csv	9.7MB
data/site4802/site4802_data2.csv	9.4MB
data/paint/paint_data1.csv	11.5MB
data/paint/paint_data2.csv	12.1MB
data/paint/paint_data3.csv	11.9MB
data/dpn/dpn_data1.csv	10.9MB
data/dpn/dpn_data3.csv	10.9MB
data/dpn/dpn_data2.csv	10.9MB
data/leer/leer_data1.csv	10.4MB
data/leer/leer_data2.csv	10.4MB
data/leer/leer_data3.csv	10.4MB
data/OSA/OSA_data1.csv	13.5MB
data/OSA/OSA_data2.csv	13.5MB
data/OSA/OSA_data3.csv	13.5MB

Each file contains 70000 rows and 19 columns. Each row contains demographic data along with simulated brain, behavior, and cognition data for each subject for a specific timepoint/wave. The column headings for each file along with a description is shown in Table 2.

**Table 2:** Data Dictionary.

Element Name	Description
subject_id	Subject Id
site_id	Site Id
Dob	Date of birth in format of ISO8601 which is YYYY-MM-DD
brain_behavior_measurement_date	This is a date associated with each timepoint/row in the database for the brain, behavior, and cognitive measurements. Each subject will have multiple time points corresponding to different waves. The format is ISO8601 which is YYYY-MM-DD
wave_number	wave number ranging from 1 to 7
autism_diagnosis	0: no, 1: yes
Age	Age in months at the time of the interview/test/sampling/imaging. Age is rounded to chronological month. If the research participant is 15-days-old at time of interview, the appropriate value would be 0 months. If the participant is 16-days-old, the value would be 1 month.
Sex	Sex of subject at birth, 0: male, 1: female
parental_education	The highest grade or level of school either parent has completed or the highest degree they have received? 0 = less than 12th grade; 1 = High school graduate or GED or equivalent Diploma; 2 = Bachelor’s degree; 3 = Master’s degree or higher; 777 = Refused to answer; use NA or blank for missing
cbcl_externalizing_raw_score	Sum of scores for externalizing section of CBCL, range 0-70
cbcl_internalizing_raw_score	Sum of scores for the internalizing section of CBCL, range 0-64
cbcl_attentionproblem_raw_score	Sum of scores for the attention problem section of CBCL, range 0-20
Iq	IQ score, general population mean 100, sd 15
wm_volume	Volume of white matter in mm^3^
gm_volume	Volume of gray matter in mm^3^
hippo_volume	Hippocampal volume in mm^3^
amygdala_volume	Amygdala volume in mm^3^
frontal_lobe_gm_thickness	Cortical thickness of frontal lobe in mm
icv	Intracranial volume in mm^3^

## Technical Validation

The data validation process involved several checks, including verifying that each required column is present in the csv files and contains the expected data type (e.g., numeric or string), confirming the total number of observations to ensure the number of rows matches the expected responses, checking that values in scale-type questions fall within the possible range, and that less than 20% of data points are missing. The range for each variable type was set such to adhere to realistic values based on the literature. A GitHub Action was used to quality check each dataset upon submission and prior to merging it with the GitHub repository. All the data files that are available in the Open Science Framework have passed the quality check. The script used for data quality check is available on https://github.com/SoCoDeN/Simulation/blob/main/tools/check_data.py.

## Data Availability

All the code used to create these datasets are available on https://github.com/SoCoDeN/Simulation/tree/main/code and all the code used to validate the datasets are at https://github.com/SoCoDeN/Simulation/tree/main/tools.
